# Subtypes analysis and prognostic model construction based on lysosome-related genes in colon adenocarcinoma

**DOI:** 10.3389/fgene.2023.1149995

**Published:** 2023-04-24

**Authors:** Yang Chen, Yunfei Lu, Changzhi Huang, Jingyu Wu, Yu Shao, Zhenling Wang, Hongqiang Zhang, Zan Fu

**Affiliations:** ^1^Department of General Surgery, The First Affiliated Hospital of Nanjing Medical University, Nanjing, Jiangsu, China; ^2^ The First College of Clinical Medicine, Nanjing Medical University, Nanjing, Jiangsu, China

**Keywords:** colon adenocarcinoma, lysosome, prognosis, tumor microenvironment, drug sensitivity

## Abstract

**Background:** Lysosomes are essential for the development and recurrence of cancer. The relationship between a single lysosome-related gene and cancer has previously been studied, but the relationship between the lysosome-related genes (LRGs) and colon adenocarcinoma (COAD) remains unknown. This research examined the role of lysosome-related genes in colon adenocarcinoma.

**Methods:** 28 lysosome-related genes associated with prognosis (PLRGs) were found by fusing the gene set that is differently expressed between tumor and non-tumor in colon adenocarcinoma with the gene set that is related to lysosomes. Using consensus unsupervised clustering of PLRGs, the colon adenocarcinoma cohort was divided into two subtypes. Prognostic and tumor microenvironment (TME) comparisons between the two subtypes were then made. The PLRGs_score was constructed using the least absolute shrinkage and selection operator regression (LASSO) method to quantify each patient’s prognosis and provide advice for treatment. Lastly, Western Blot and immunohistochemistry (IHC) were used to identify MOGS expression at the protein level in colon adenocarcinoma tissues.

**Results:** PLRGs had more somatic mutations and changes in genetic level, and the outcomes of the two subtypes differed significantly in terms of prognosis, tumor microenvironment, and enrichment pathways. Then, PLRGs_score was established based on two clusters of differential genes in the cancer genome atlas (TCGA) database, and external verification was performed using the gene expression omnibus (GEO) database. Then, we developed a highly accurate nomogram to enhance the clinical applicability of the PLRGs_score. Finally, a higher PLRGs_score was associated with a poorer overall survival (OS), a lower tumor mutation burden (TMB), a lower cancer stem cell (CSC) index, more microsatellite stability (MSS), and a higher clinical stage. MOGS was substantially elevated at the protein level in colon adenocarcinoma as additional confirmation.

**Conclusion:** Overall, based on PLRGs, we identified two subtypes that varied significantly in terms of prognosis and tumor microenvironment. Then, in order to forecast patient prognosis and make treatment suggestions, we developed a diagnostic model with major significance for prognosis, clinical relevance, and immunotherapy. Moreover, we were the first to demonstrate that MOGS is highly expressed in colon adenocarcinoma.

## Introduction

Cancer incidence and mortality, which is second only to heart disease (Siegel et al., 2021), are increasing rapidly around the world, with colorectal cancer accounting for about 9% of the total (Bray et al., 2018). In recent years, the number of colorectal cancer cases and deaths in China has also increased (Li et al., 2021a), and colon adenocarcinoma, the main component of colorectal cancer, has received increased attention (Siegel et al., 2020). Despite the availability of numerous treatments, including endoscopic therapy, surgical treatment, radiotherapy, immunotherapy, and targeted therapy, the 5-year OS time for patients with COAD is still dismal. Patients with local tumor spread have a 5-year OS time of 69.2%, while patients with distant metastasis have a 5-year OS time of only 11.7% (Brenner et al., 2014). Traditional histological classification approaches, however, cannot adequately direct the treatment of all patients due to the high heterogeneity of colonic malignancies (Punt et al., 2017). In light of this circumstance, an increasing number of studies have classified patients into several prognostic subtypes. Dai et al. divided colorectal cancer patients into two prognostic subtypes based on senescence-related genes (Dai et al., 2022). Based on genes related to lipid metabolism, Jiang et al. divided COAD patients into three subtypes with distinct prognostic characteristics (Jiang et al., 2021). Hence, to provide more suggestions for further treatment, we wanted to use new multiple molecules to construct molecular subtypes of COAD and construct a prognostic model to quantify the prognosis of COAD patients and provide recommendations for subsequent treatment.

The lysosome, an organelle that digests both endocytic extracellular material and autophagic intracellular material, is the cell’s primary degradation site (Piao and Amaravadi, 2016). Lysosome dysfunction influences the onset and progression of diseases such as cancer (Ballabio and Bonifacino, 2020). Previous research has found that lysosomes are linked to a number of cancers. By activating the AKT signaling pathway, lysosomes promote cancer progression and metastasis (Radisavljevic, 2019). The activation of the lysosomal clearance system is a new marker for assessing pancreatic cancer invasiveness (Perera et al., 2015). In mice, AMPK-mediated lysosomal function promotes the development of lung cancer (Patra et al., 2019). HSPA5 was found to be a protective factor in HNSCC by maintaining lysosomal activity (Kim et al., 2018). LRGs have been previously studied (Haratake et al., 2021; Li et al., 2022), LAPTM5 is a potential diagnostic marker for hypertensive left ventricular hypertrophy, and SLCA38A7 overexpression in lung cancer represents a poor prognosis. Significant progress has also been made in the study of LRGs recently (Pechincha et al., 2022; Richards et al., 2022). LYSET, a molecule that allows cancer cells to feed on extracellular proteins, was found to be required for the mannose 6-phosphate (M6P) lysosomal transport pathway. Another study published around the same time found that inhibiting LYSET decreased the efficiency of lysosomal transport and tumor progression was slowed. These findings have revealed a link between lysosomes and diseases, particularly cancer. These studies, however, were limited to a single gene, and no study based on all LRGs has been established. Hence, subtypes based on LRGs were constructed, and prognostic model was constructed by DEGs between the two subtypes.

We performed a series of systematic analyses after intersecting LRGs with the differentially expressed genes (DEGs) between tumor and normal in COAD from TCGA database. First, we used consensus unsupervised clustering analysis to divide patients into two clusters based on the PLRGs expression levels, and then prognostic, TME and pathway enrichment analyses were performed between the two clusters. After which, to further investigate the role of the PLRGs in COAD, we built a prognostic model based on the DEGs between the two clusters. We evaluated the prognosis, clinical relevance, and immunotherapy of the TCGA group using the prognostic model, and we used the GEO database for external validation. These findings demonstrated that a novel multi-molecule diagnostic model based on PLRGs can evaluate the prognosis of COAD and provide further treatment recommendations.

## Materials and methods

### Data source

Our study included a total of 588 COAD patients from various platforms. The TCGA data portal was used to obtain raw genotype data for COAD patients, including RNA-seq transcriptome data (fragments per kilobase million, FPKM) and related clinical and survival information (Supplementary Table S1). The TCGA-COAD cohort served as the training cohort in this study, while the GSE17538 cohort from the GEO database served as the independent validation cohort. Simultaneously, we searched the Gene Ontology (GO) database for the 876 LRGs. To eliminate batch effects between the two data sets, the “Combat” package was used. To reduce bias, we also excluded patients with the OS time of less than 30 days. Following that, copy number variation (CNV) files and somatic mutation data were obtained using the TCGA COAD cohort. Using the “DESeq2” package, the differentially expressed genes between normal samples and tumor samples from the TCGA database were examined. The web-based gene network prediction tool GeneMANIA was used to create protein interaction networks.

### Consensus clustering analysis of PLRGs and relationships between different subtypes

Using univariable Cox regression (*p* < 0.05), we chose 28 genes strongly related to prognosis from the group of genes linked to the lysosomes. In order to categorize patients into several subtypes based on the levels of the PLRGs expression, consensus unsupervised clustering analysis was carried out using the R “ConsensusClusterPlus” package. To keep the clustering consistent, we went through 1,000 iterations. Next, the correlation between clinicopathological characteristics and prognosis and molecular subtypes was assessed. In addition, using Kaplan-Meier (KM) analysis and the accompanying “survival” and “survminer” packages (Rich et al., 2010), this article compared the OS and the disease-free survival (DFS) of the two subtypes. Furthermore, R software’s gene set variation analysis (GSVA) was employed to evaluate variations in biological pathways (Hänzelmann et al., 2013) among the two subtypes. The immunological score and stromal score for each patient were then calculated using the ESTIMATE method to explore for variations between the immune microenvironment of different subtypes (Meng et al., 2020). By then, we had performed single-sample gene set enrichment analysis (ssGSEA) to calculate the degree of immune cell infiltration among different subtypes (Huang et al., 2021). Finally, the differences in immune checkpoint genes and immune activation genes between the two subtypes were discussed.

### DEGs identification and functional annotation

The R package “limma” was applied to recognize DEGs between the two clusters (FDR<0.05 and log2 fold change≥1). Functional enrichment analysis on the DEGs was performed using the R package “clusterprofiler” to explore the probable activities of clusters-associated DEGs and find related gene functions and enriched pathways (Yu et al., 2012).

### Construction of the prognostic PLRGs_score and nomogram

To furtherevaluate the value of the two subtypes of DEGs for COAD prognosis, the paper employed the TCGA COAD cohort as the training group and the GSE17538 cohort as the external validation group. Univariate Cox regression was implemented in the TCGA group for differential genes (*p* < 0.001), and the univariate Cox results were then reduced in dimensionality using LASSO regression. For the final screening, multivariate Cox regression was used, and seven genes were obtained.

$$PLRGs\_score=\sum_{n=1}^{i}\left(Coefi\;*\;PLRGsExp\right)$$

The coefficient and expression levels of the relevant genes are represented, respectively, by the Coefi and PLRGsExp. Depending on the median risk score, all samples were split into low-risk (PLRGs_score < median value) and high-risk (PLRGs_score > median value) subgroups. The differences in OS between the various risk subgroups were examined using KM analysis. The “survivalROC” R package was used to create the time-dependent receiver operating characteristic (ROC) curve that measures PLRGs_ score’s accuracy. Both univariate and multivariate Cox analyses showed that the PLRGs_score had a substantial impact on all clinical features. Finally, utilizing the “rms” software, a nomogram was developed to forecast 1-, 3-, and 5-year survival. The correctness of the nomogram was evaluated by means of calibration and ROC. Results were assessed in a group that underwent external validation (GSE17538).

### Evaluation of TME in lysosome-related signature

First, the seven lysosome-related signature genes and risk scores were explored for their links to immune cells. In order to compare the immune infiltration of the two groups based on the PLRGs_score, the paper next calculated the ESTIMATE score and examined the differential distribution of ssGSEA based on 22 immune cells between two groups. Finally, the distinctions between the two subtypes’ immune checkpoints and immune activation genes were explored.

### Mutation, microsatellite instability (MSI), TMB and CSC

The somatic mutations of the COAD patients were divided into high- and low-risk groups using the mutation annotation format (MAF) generated from the TCGA database using the “maftools” R package (Mayakonda et al., 2018). For every COAD patient in the two groups, we also calculated the immunophenotype score (IPS) and TMB scores. And we also took into account the linkages between the two risk groups in addition to CSC and MSI.

### Drug susceptibility and clinical correlation analysis

Using chi-square testing, the relationships between the PLRGs_score and the clinical characteristics (age, sex, stage, TNM stage) were examined. In order to further evaluate variations in the therapeutic effects of chemotherapeutic medications in the two subgroups, we computed the semi-inhibitory concentration (IC50) values of chemotherapeutic agents routinely used to treat COAD patients using the “pRRophetic” program (Geeleher et al., 2014).

### Collection of clinical samples

Samples of human COAD and nearby normal mucosa were taken from COAD patients who had surgery at the First Affiliated Hospital of Nanjing Medical University between 2014 and 2018. From a previous study, specific information about the samples could be obtained (Shen et al., 2021). The First Affiliated Hospital of Nanjing Medical University’s Ethics Committee approved all of the experiments, and each patient gave their informed consent before participating in this study.

### Western blot analysis

Using the total protein extraction kit (RS0024, Immunoway, China), the total protein samples from the tissues were extracted in accordance with the manufacturer’s instructions. To measure the total protein, a BCA Protein Assay kit (P0012, Beyotime, Shanghai) was used. SDS-PAGE gel with a 10% concentration was used to transfer the protein samples to nitrocellulose (NC) membranes (HATF00010, Millipore, Shanghai). These membranes were treated with the MOGS primary antibody (1:1000; 17859-1-AP, Proteintech, Hubei) overnight at 4°C after being blocked for 60 min with 5% skim milk powder. The HRP-conjugated Affinipure Goat Anti-Rabbit IgG (1:10000; SA00001-2, Proteintech, Hubei) was then incubated on the membrane for 60 min at 37°C before the proteins were detected. Also, the straps’ gray-scale values were detected using ImageJ software for quantitative analysis.

### Immunohistochemistry (IHC)

The 18 pairs tissue sections were rehydrated with an alcohol gradient after being deparaffinized with xylene for IHC examination. For 10 min at room temperature, the samples were exposed to 3% H2O2 to inhibit endogenous peroxidase activity. The samples were put in an EDTA buffer for antigen retrieval (PH 9.0; AFIHC010, Aifang, Hunan). The samples underwent an overnight incubation at 4°C with the MOGS antibody (1:100; 17859-1-AP, Proteintech, Hubei), followed by a 30-minute incubation at room temperature with the IHC antibody kit (AFIHC001, Aifang). After applying the DAB chromogenic kit (AFIHC004, Aifang) to the samples, the nuclei were counterstained with hematoxylin. Finally, a digital microscope camera was used to record each sample image (AE41, Motic). The percentage of stained samples that were positive was calculated as 0 = negative, 1 = < 10%, 2 = 10%–49%, and 3 = ≥ 50%. According to the intensity scale, 0 indicates no staining, 1 is little, 2 is moderate, and 3 is strong (Karpathiou et al., 2021). The percentage and intensity were multiplied to determine the final results for each section.

### Statistical analysis

To find the difference between the two groups, the Wilcoxon rank-sum test was performed. To ascertain if the clinicopathological indicators may be employed as independent prognostic factors, univariate and multivariate Cox regression were used. Every experimental result was displayed as Mean ± SD (standard deviations). All statistical evaluations were performed in R 4.1.2 and GraphPad Prism 9. Statistical significance was set at *p* < 0.05.

## Results

### Screening of the PLRGs and description of the genetic mutations

**FIGURE 1:**
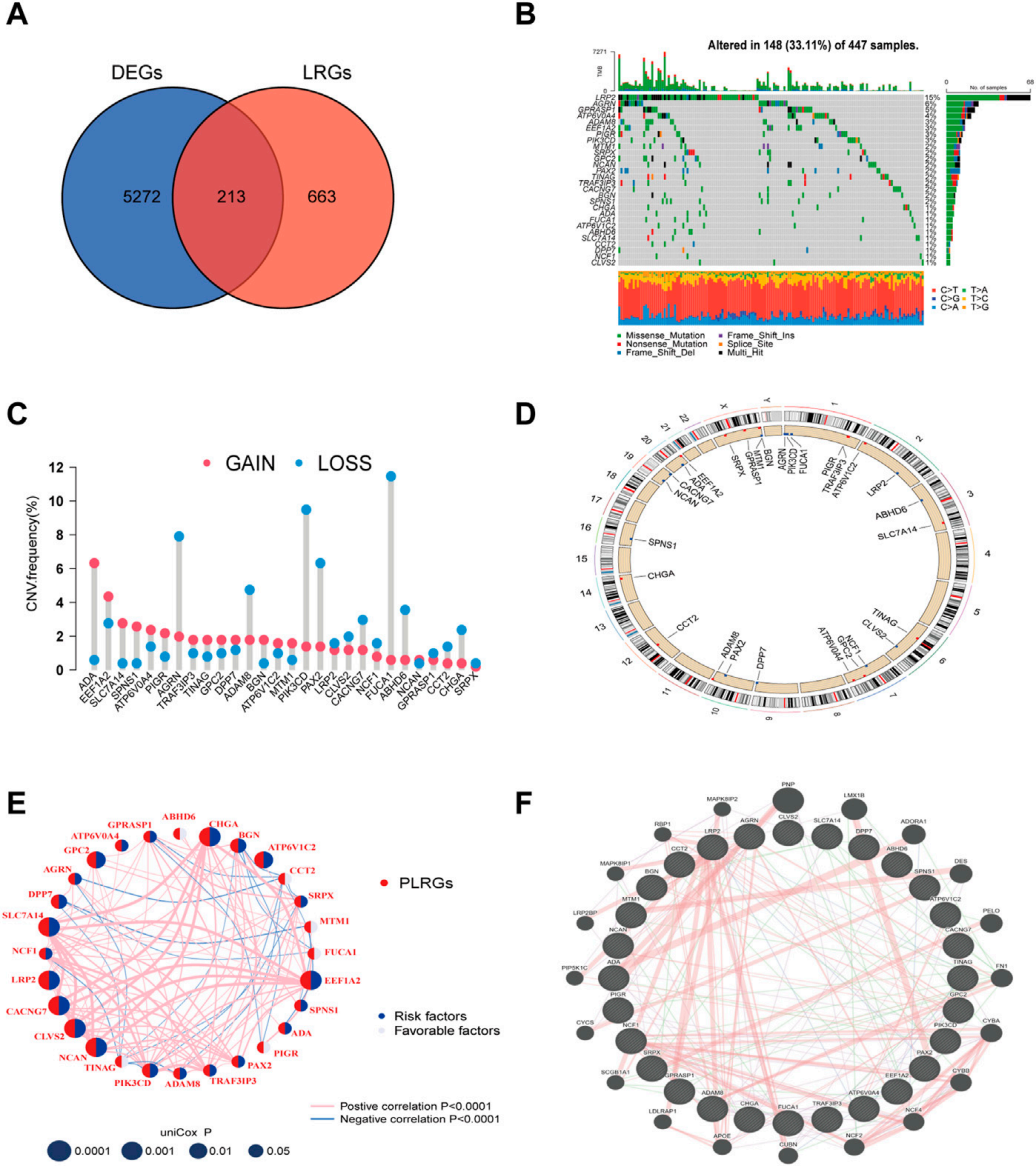
LRGs screening and PLRGs genetic mutations. **(A)** Intersection of tumor and non-tumor DEGs and LRGs. **(B)** Genetic variation in 28 PLRGs. **(C)** Frequency of CNV in 28 PLRGs. **(D)** PLRGs distribution across 23 chromosomes. **(E)** Correlation network of 28 PLRGs in the TCGA cohort. **(F)** Gene-gene interaction of 28 PLRGs predicted by GeneMANIA.

First, 456 tumor and 41 normal samples obtained from the TCGA-COAD cohort were subjected to differential gene analysis (log FC > 1, *p*-value <0.05). This process identified 5485 DEGs. Then, using the GO database, 876 LRGs were collected. Finally, 213 LRGs in total were intersected (Figure 1A). The Cox regression method was then used to test these genes for prognosis-related genes. The univariate Cox regression screening cutoff was *p* < 0.05, and 28 genes with related HR values were discovered (Supplementary Table S2). Then, 28 PLRGs in the COAD cohort were examined for the presence of CNVs and somatic mutations. As shown in Figure 1B, 148 (33.11%) samples had mutations in the TCGA cohort. The most often mutated of the genes was LRP2, followed by ARGN and GPRASP1. All 28 PLRGs can be shown to have copy number changes in Figure 1C. On each chromosome, 28 PLRGs were mapped according to their position (Figure 1D). The extensive landscape of 28 PLRGs interactions, regulator interconnections, and clinical outcome in COAD patients were clearly displayed by the PLRGs network (Figure 1E). The network of the 28 PLRGs and the 20 most frequently altered neighboring genes was also built (Figure 1F).

### Establishment of subtypes based on PLRGs

**FIGURE 2:**
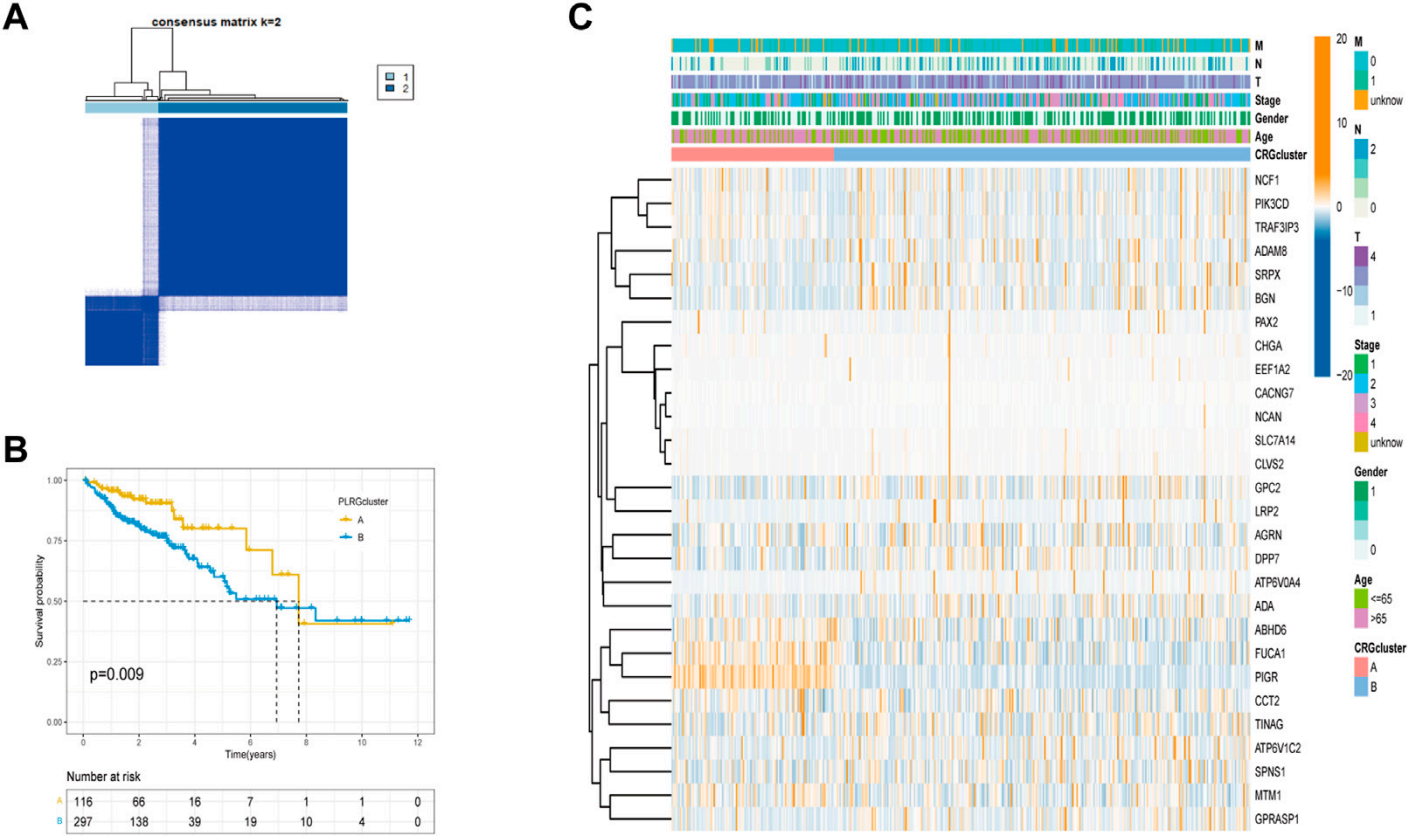
Tumor classification based on PLRGs. **(A)** The consensus clustering matrix (*k* = 2) was used to divide COAD patients in the TCGA group into two subtypes. **(B)** A KM analysis of the two clusters was performed. **(C)** Clinicopathological features and gene expression heatmaps for the two clusters.

**FIGURE 3:**
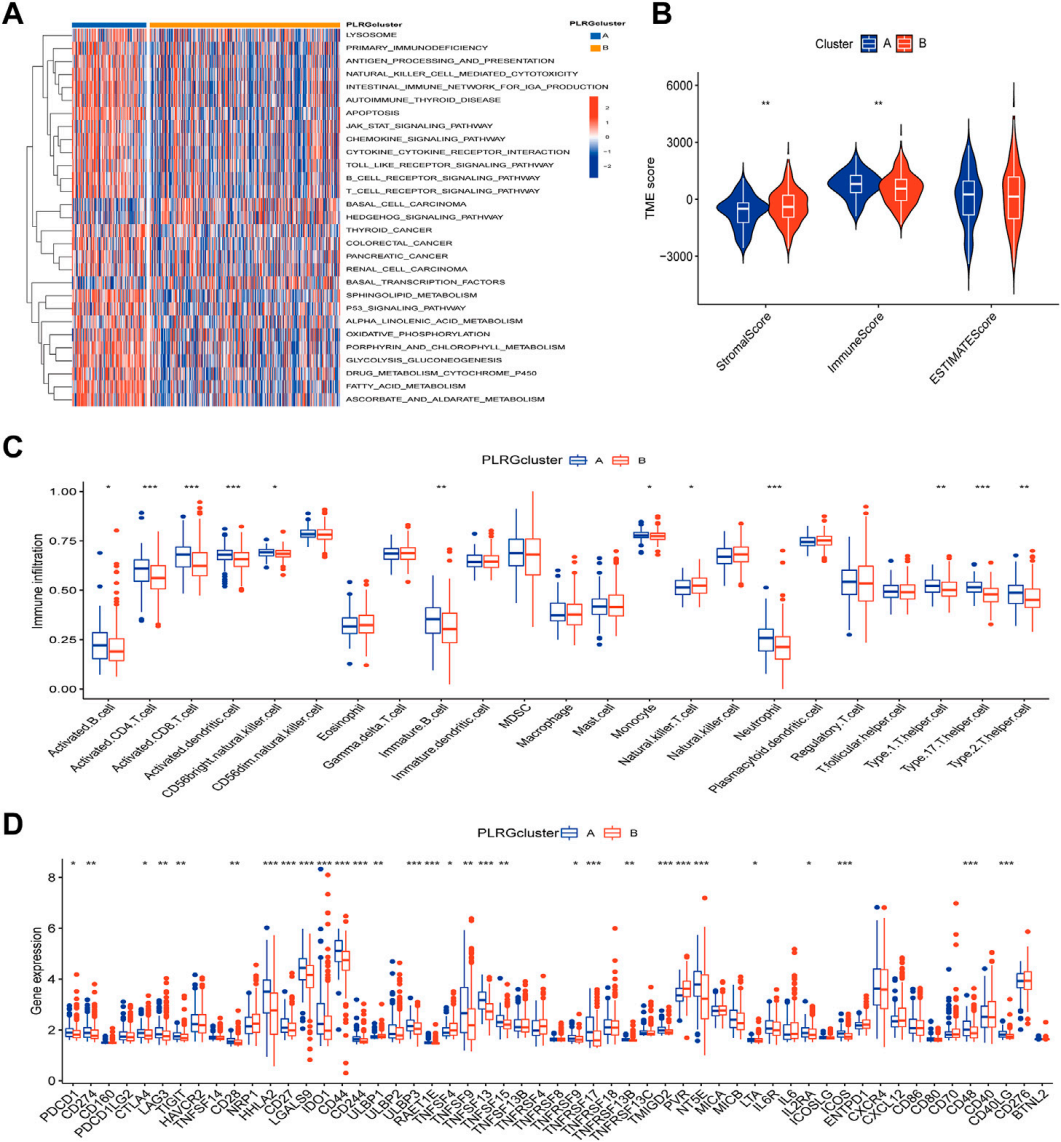
Differences in tumor immune microenvironment between the two clusters. **(A)** KEGG pathway enrichment analysis between two clusters. **(B)** Estimate algorithm between two clusters. **(C)** The infiltration of 23 immune cells between the two clusters was analyzed using the ssGSEA algorithm. **(D)** Differences expression in immune checkpoint and immune activation-genes between the two clusters. (*p* < 0.05 *; *p* < 0.01 **; *p* < 0.001 ***).

To further investigate the relationship between PLRGs and COAD, we employed a consensus clustering analysis to divide TCGA-COAD patients into two clusters (Cluster A: n = 116, Cluster B: n = 297; Figure 2A, Supplementary Figure S1A-H). The two clusters had a significant difference in OS time (*p* = 0.009; Figure 2B). And, the two clusters had a significant difference in DFS (*p* = 0.012; Supplementary Figure S1I). Heatmaps of clinical information were connected between the two clusters, as seen in Figure 2C. Finally, we investigated differences in biological function using GSVA enrichment analysis. Cluster A was abundant in cancer-associated pathways such as colorectal cancer, thyroid cancer, pancreatic cancer, renal cell carcinoma, P53 signaling pathway, AK STAT signaling pathway, and apoptosis, according to the findings of the GSVA analysis. T cell receptor signaling pathway, B cell receptor signaling pathway, natural killer cell mediated cytotoxicity, Toll like receptor signaling pathway, and chemokine signaling pathway were also abundant in Cluster A (Figure 3A). Surprisingly, cluster A also enriched a variety of metabolic pathways.

### Analysis of the TME of the two subtypes in COAD

Since the results of GSVA analysis showed more enrichment of immune-related pathways, we performed ESTIMATE analysis, ssGSEA analysis, and an analysis of the differences in the expression of immune-related genes between the two clusters to better understand the TME differences between the two clusters. We can see that there were significant differences in StromalScore and ImmuneScore between the two groups (Figure 3B). Most immune cell infiltrations were found to differ significantly between the two clusters using ssGESA analysis (Figure 3C). Cluster A was dominated by activated B cells, activated CD4 T cell, activated CD8 T cell and so on, while Cluster B was dominated by Natural killer T cells. Finally, in the difference analysis of immune checkpoint and immune activation related genes (Figure 3D), for example, PDCD1, CD274, and CTLA4 were highly expressed in Cluster A, whereas Cluster B only had high levels of TNFSF4.

### DEGs identification and construction of the prognostic model

**FIGURE 4:**
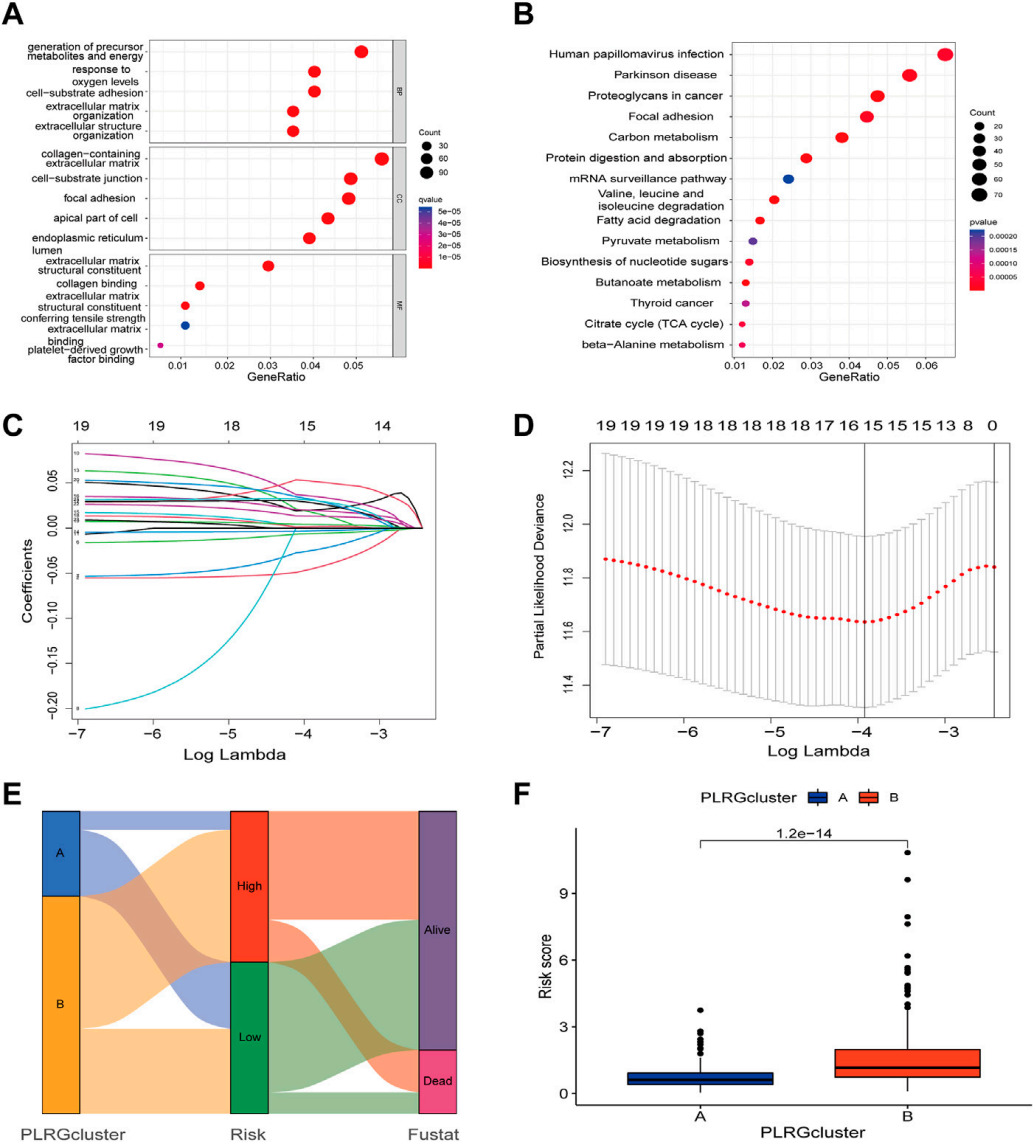
Construction of a prognostic model based on differential genes between the two clusters. **(A,B)** GO/KEGG analysis between the two clusters; **(C)** LASSO regression based on differential genes between the two clusters. **(D)** Cross-validation of the LASSO model based on 7 genes. **(E)** The Sankey diagram with clusters, prognostication models, and outcomes for survival. **(F)** Differences in risk scores between the two clusters.

The above analyses revealed significant differences in clinical prognosis, signaling pathways, and tumor microenvironment between the two clusters. The two clusters were analyzed for differential genes for further study, and 2,129 differential genes were eventually obtained (Supplementary Table S3). The obtained differential genes were then analyzed for GO/KEGG enrichment (Figures 4A, B). Simultaneously, differential genes were used in the following analysis. Univariate Cox analysis was used to identify 23 genes associated with prognosis (Supplementary Table S4), followed by Lasso analysis to identify 15 genes (Figures 4C, D), and finally, the multivariate Cox algorithm was used to identify 7 genes (Supplementary Table S5), from which the prognosis model was built.

Risk score = (−0.08209* expression of ZDHHC3) + (−0.01682* expression of GSR) + (0.08702* expression of HEYL) + (0.053025* expression of TRIP10) + (0.059997* expression of MOGS) + (0.052277* expression of UCHL1) + (0.017473* expression of PODXL).

Figure 4E showed the connection between clusters, risk, and survival state. As can be seen from prior studies showing that Cluster A had a better prognosis than Cluster B, Figure 4F demonstrated substantial differences between the two clusters, with Cluster A having a lower risk score.

### Survival analysis in TCGA group and validation in the GEO group

**FIGURE 5:**
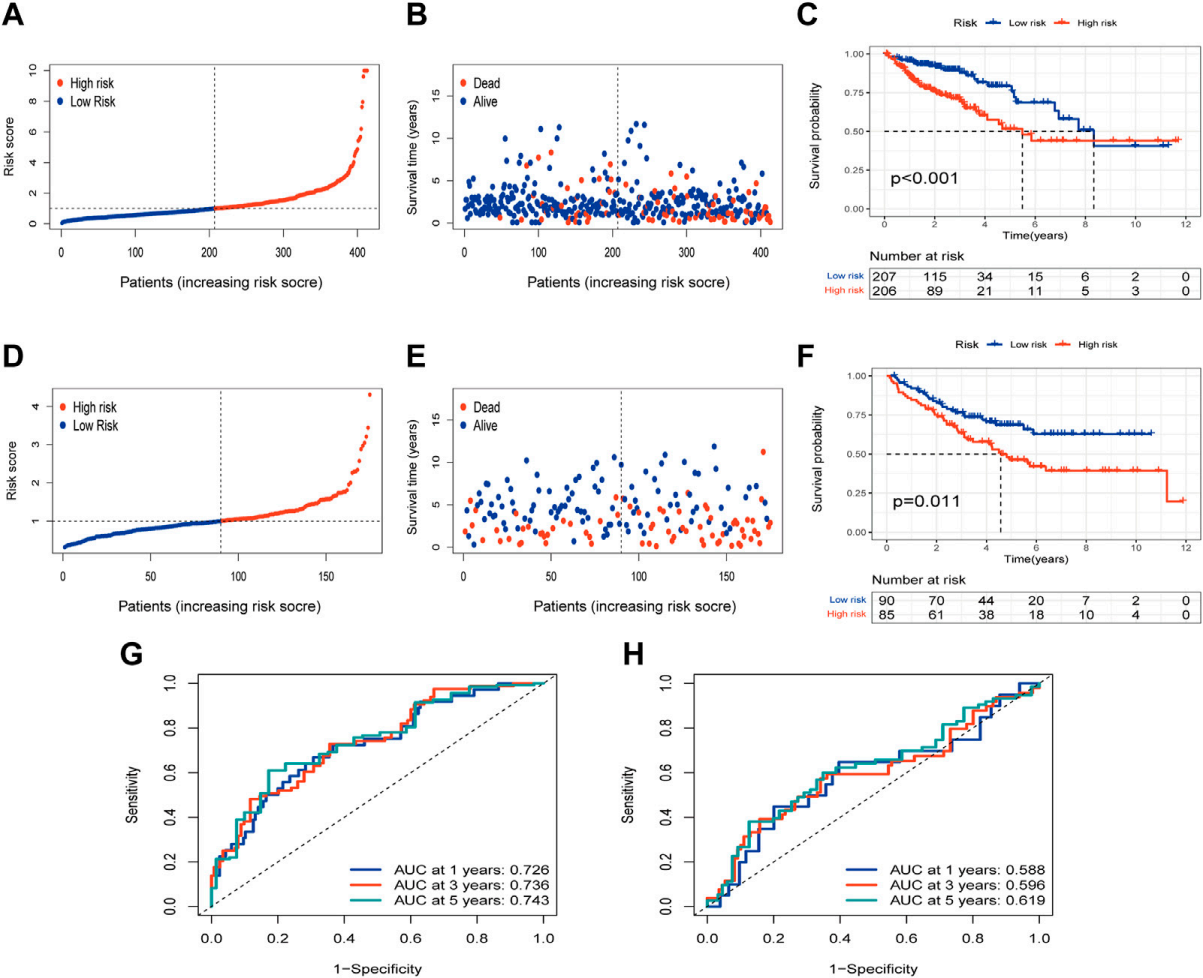
Examination of the prognostic model in the TCGA database and external validation of the model in the GEO database. **(A,B)** Distribution and survival of each patient in the TCGA group. **(C)** OS time difference between the two groups in the TCGA group. **(D,E)** Distribution and survival of each patient in the GEO group. **(F)** OS time difference between the two groups in the GEO group. **(G)** Time-dependent ROC curves in the TCGA group. **(H)** Time-dependent ROC curves in the GEO group.

The risk of PLRGs_score distribution plot in the TCGA group revealed that as PLRGs_score increased, survival time decreased (Figures 5A, B). The KM survival curves showed that patients with high scores had a considerably shorter OS duration than individuals with low scores (Figure 5C). Furthermore, AUC values of 0.726, 0.736, and 0.743 were used to represent the 1-, 3-, and 5-year survival rates of PLRGs_score, respectively (Figure 5G). The same findings were observed in the external validation group, with patients with a higher risk score having a worse prognosis (Figures 5D–F). And AUC values of 0.588, 0.596 and 0.619 were used to represent the 1-, 3-, and 5-year survival rates of PLRGs_score in the validation group (Figure 5H).

### Construction of a nomogram

**FIGURE 6:**
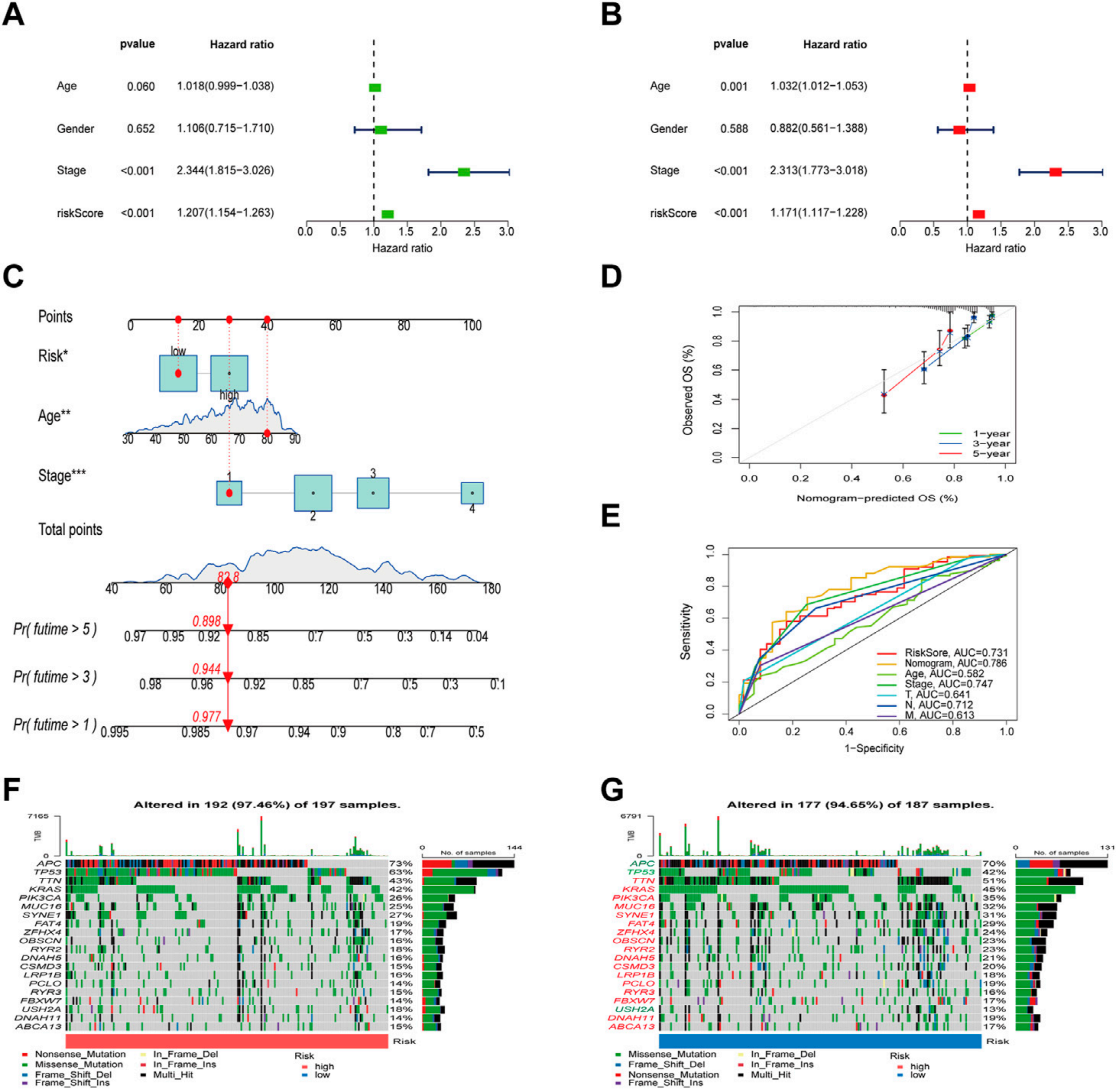
Evaluation of the risk model and the mutation difference between the two groups. **(A,B)** Analysis of risk scores and clinical information using univariate and multivariate Cox analyses. **(C)** Establishment of a nomogram for OS prediction. **(D)** Calibration curves for the 1-, 3-, and 5-years OS. **(E)** The ROC curves of the nomogram. **(F,G)** Waterfall diagram of mutations between high and low risk groups.

Univariate and multivariate Cox analysis were conducted to test if the model’s scores could be utilized as independent prognostic indicators, and the findings revealed that risk scores were capable of serving as independent prognostic variables for COAD patients (Figures 6A, B). We then calculated the 1-, 3-, and 5-year OS time for these patients based on age, stage, and risk (Figure 6C). The calibration curves of this well-established nomogram showed excellent concordance between observed reality and anticipated values (Figure 6D). Additionally, we calculated these clinical parameters’ AUC values for predicting OS time (Figure 6E), as well as the AUC values of stage and TNM stage for 1-, 3- and 5- years, respectively (Supplementary Figure S2).

### Association of PLRGs_Score with mutation, TMB, MSI, and CSC score

**FIGURE 7:**
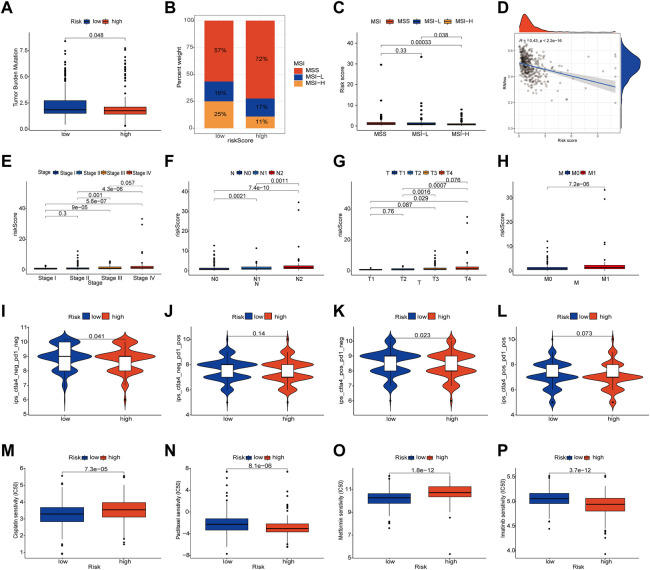
Multifaceted analysis between high and low risk groups. **(A)** Relationships between PLRGs_score and TMB. **(B,C)** Relationships between PLRGs_score and MSI. **(D)** Relationships between PLRGs_score and CSC index. **(E–H)** Clinical stage and TNM stage in different PLRGs_score groups. **(I–L)** IPS in different PLRGs_score groups. **(M–P)** Relationships between PLRGs_score and drug sensitivity.

To check for variations in the distribution of COAD patients’ somatic mutations, we first searched the TCGA database. While the mutation rates of the other genes were higher, it was discovered that the mutation rates of APC, TP53, and USH2A were lower in the low-risk group as compared to the high-risk group (Figures 6F, G). Numerous studies have demonstrated the value of TMB and MSI as indicators of the tumor immune response and the therapeutic potential of ICP inhibitors in patients with high TMB or MSI. We thus performed TMB and MSI investigations. The TMB of the high-risk group was lower than that of the low-risk group, as shown in Figure 7A, suggesting that the low-risk group may have had a higher degree of immunotherapy efficacy. Significant differences existed between MSS and MSS-H, as well as between MSS-H and MSS-L, but no difference existed between MSS and MSS-L (Figure 7C). The low-risk group may respond better to subsequent immunotherapy since the high-risk group had a higher proportion of MSS while the low-risk group had a higher proportion of MSS-H (Figure 7B). Then, in order to assess their possible importance in COAD, we merged the PLRGs_score and CSC scores (Figure 7D). There was a substantial negative association between the risk scores and the CSC scores, showing that the lower the risk scores, the more significant the stem cell properties of COAD cells were and the lower the degree of cell differentiation.

### Clinical correlation and drug sensitivity analysis

Clinical staging is currently the world’s most authoritative tumor staging method, and we want to see if the risk score obtained in this study is related to clinical staging. Consequently, an examination of the connection between the risk score and the Stage and TNM stage was carried out. Significant differences existed between Stage 1&2 and Stage 3&4, T1&2 and T3&4, N1, N2 and N3, and M0 and M1, and poorer clinical grades were positively connected with greater risk ratings (Figures 7E–H). We also investigated the relationship between risk scores and age and gender, but no differences were discovered (Supplementary Figure S3). Following that, the IPS was used to validate our hypothesis by assessing how colon cancer patients responded to immunotherapy pairs. When the high-risk group did not respond well to PD1 therapy and when CTLA4 was either positive or negative, there was a substantial difference between them and the low-risk group. However, the other conditions were not statistically significant (Figures 7I–L). Finally, we ran a drug sensitivity analysis in COAD, calculated the IC50 values for each drug, and selected four representative drugs to display. The high-risk group responded better to Cisplatin and Metformin, as seen in the figure. Imatinib and Paclitaxel both performed well in the low-risk group (Figures 7M–P).

### TME discrepancies between the high- and low-risk groups

**FIGURE 8:**
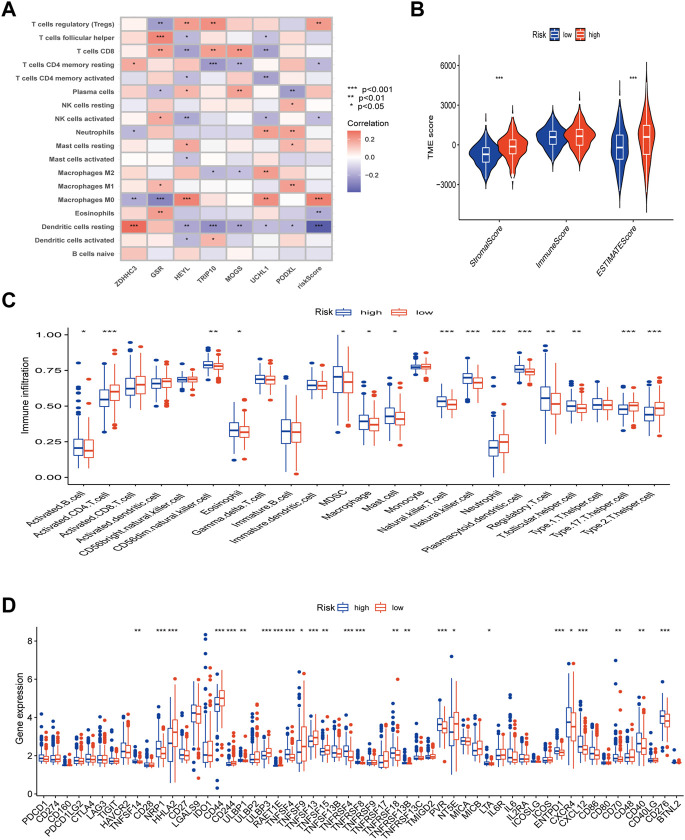
TME, immune checkpoints and immune activation-related genes analysis of the risk model. **(A)** Correlations between the abundance of immune cells and 7 genes and risk scores. **(B)** Differences between both immune and stromal scores in the two groups. **(C)** The infiltration of 23 immune cells between the high and low risk groups. **(D)** Differences expression in immune checkpoints and immune activation-related genes between the high and low risk groups. (*p* < 0.05 *; *p* < 0.01 **; *p* < 0.001 ***).

First, we looked into the relationship between immune cell enrichment, seven genes, and risk scores in the model. Figure 8A illustrated the significant correlation between the 7 genes and risk scores and immune cell enrichment. The risk score was mostly related to Tregs, T cells with resting CD4 memory, activated NK cells, Macrophages M0, and resting dendritic cells. The two groups’ StromalScore and ESTIMATEScore, as determined by ESTIMATE analysis, significantly differed from one another (Figure 8B). After examining the risk ratings for ssGSEA enrichment, most immune cells displayed appreciable enrichment differences between the high and low risk groups (Figure 8C). Finally, employing immune checkpoint and immune activation-related genes, researchers found that the majority of immunological activation-related genes were significantly expressed differently between the high and low risk groups. Unfortunately, the bulk of immune checkpoint genes were similar in both groups (Figure 8D).

### Detection of MOGS expression in colon adenocarcinoma

**FIGURE 9:**
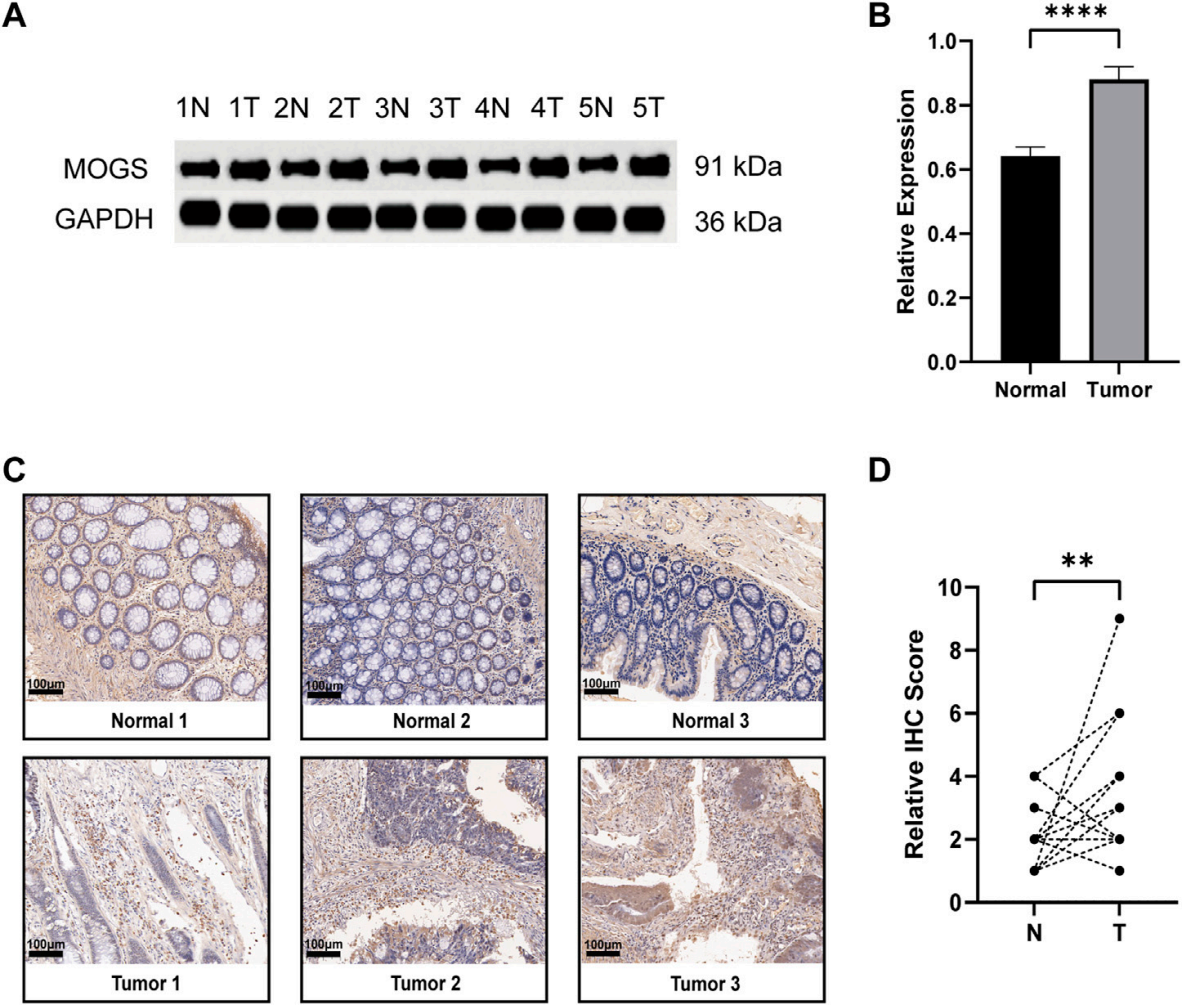
Expression of MOGS at the protein level in COAD. **(A)** Protein level of MOGS detected by Western Blot in 5 matched primary tumor and adjacent normal tissues. **(B)** The relative expression of MOGS protein levels in COAD tissues. The histogram indicated the signal intensity of the proteins against GAPDH (mean ± SD, *n* = 5, *p* < 0.0001 ****). **(C)** Representative IHC staining of MOGS in 3 COAD tissues and paired adjacent normal tissues. Scale bar, 100 μm. **(D)** Relative IHC score. (*n* = 18, *p* < 0.01 **).

Except for MOGS, all six of the prognostic model’s genes have been investigated previously in cancer. As a result, we were able to confirm for the first time the link between MOGS and COAD. First, we chose 5 pairs of COAD tissues and normal tissues and then used Western Blot to confirm that MOGS was highly expressed in tumor tissues compared to normal tissues at the protein level (Figures 9A, B). IHC was then used to show that MOGS was highly expressed in tumor tissues (Figures 9C, D).

## Discussion

Lysosomes are centres of signaling and cell breakdown that are essential for cell growth, senescence, and homeostasis (Yang and Wang, 2021). Increasing evidence points to the role of lysosomes in the development, occurrence, and recurrence of cancer. Since lysosome-mediated programmed cell death in cancer cells is achieved by lysosome disruption (Kreuzaler and Watson, 2012), lysosomes may offer new therapeutic options for the treatment of cancer progression brought on by apoptosis (Aits and Jäättelä, 2013). In addition to cell death, lysosomes now have some new roles in tumor growth, invasion, and metastasis (Ballabio and Bonifacino, 2020). Lysosomal autophagy has also been linked to prostate cancer, glioblastoma, pancreatic cancer, and lung cancer, according to earlier research (Kimmelman and White, 2017). And lysosomes in COAD have also been extensively researched. For instance, COAD tissues have elevated expression levels of the lysosome associated membrane proteins 1 and 2 (LAMP-1 and LAMP-2) (Furuta et al., 2001). LAPTM4B genetic variation may increase the chance of developing COAD (Cheng et al., 2008). After ATP6V0E2 is knocked down, anlotinib’s capacity to trigger lysosome function is reduced, which leads to apoptosis of colon tumor cells (Sun et al., 2020). As previously stated, single lysosome-related gene have been widely concerned and studied, however, we focus on multiple LRGs and associate them with COAD in order to investigate the subtype analysis of multiple LRGs in COAD and the development of a prognosis model.

Prior to selecting genes for unsupervised clustering, the researchers crossed LRGs whose expression varied across tumor and normal tissues. The clustering results revealed that among other things, there were significant differences between the two subtypes in terms of prognosis, tumor microenvironment, immunological checkpoint, and enrichment pathway. Cluster A had a better prognosis than Cluster B, and more immune-related genes were expressed there. In addition, pathways related to immunology, metabolism, and malignancies were shown to be enriched, according to the GSVA enrichment research. Based on the aforementioned results, a lasso prognostic model was created using the difference genes between the two subtypes. We discovered that the high-risk and low-risk groups had significantly different overall survival times, with the high-risk group having a worse prognosis. Additionally, Cluster A’s lower risk score is in line with its earlier improved prognosis. Using univariate and multivariate Cox regression, it was then demonstrated that this risk score was an independent predictive predictor for colon cancer. We created nomograms based on the Cox results, and ROC curves confirmed the accuracy of the 1-year, 3-year, and 5-year OS projections.

Our prognostic signature consists of seven genes: Previous studies on cervical and breast cancer found that ZDHHC3, Zinc Finger DHHC-Type Palmitoyltransferase 3, was highly expressed in cervical cancer (Choi et al., 2007) and showed copy number amplification after HPV infection (Li et al., 2021b). ZDHHC3 inhibits breast cancer cell growth while also promoting oxidative stress and aging (Sharma et al., 2017). Simultaneously, inhibiting the expression of this molecule can improve the immune response of T cells to tumors (Yao et al., 2019). GSR, Glutathione-Disulfide Reductase, has been extensively researched in a variety of cancers. According to a recent study (Wang et al., 2020), AMPK1 promotes the occurrence of colorectal cancer by regulating GSR phosphorylation. By inhibiting this gene, it has been demonstrated that HEYL functions as an interaction between the TGF- and Notch signaling pathways (Han et al., 2014). And it has also been shown to promote tumor metastasis in lung cancer (Wang et al., 2021) and may also promote metastasis in colorectal cancer (Weber et al., 2019). TRIP10, also known as CIP4, is controlled by AKAP9 and aids in the development of colorectal cancer (Hu et al., 2016). It had been demonstrated that colorectal cancer enhanced high levels of TRIP10 expression. At the same time, it promotes tumor metastasis in cancers like nasopharyngeal cancer, breast cancer, lung cancer, and others (Rolland et al., 2014; Cerqueira et al., 2015; Meng et al., 2017). UCHL1, Ubiquitin C-Terminal Hydrolase L1, which has been extensively studied in tumors such as lung and breast cancer as well as colorectal cancer (Yang et al., 2022), plays a catalytic role in colorectal cancer (Zhong et al., 2012). PODXL, whose overexpression promotes pancreatic cancer development (Taniuchi et al., 2022); TGF and its mediated PODXL, like the previously mentioned genes, play corresponding roles in colorectal cancer. Patients with radiotherapy-resistant colorectal cancer may benefit from treatment interventions targeting TGF inhibition and PODXL activation (Lee et al., 2021). The preceding chart summarized the progress of six genes in cancer research, with a focus on colorectal cancer. MOGS, or Mannosyl-Oligosaccharide Glucosidase, was rarely mentioned in cancer research. As a result, we performed Western Blot and IHC experiment on it. For the first time, we investigated MOGS expression in COAD, and the findings demonstrated that MOGS was substantially more expressed in tumor tissues than in adjacent normal tissues.

Then, we performed several analyses utilizing two groups with high and low risk scores. Cancer development is linked to mutations in cancer cell genes (Iranzo et al., 2018). Because of this, we performed a tumor mutation load analysis. The results demonstrated that a lower TMB in the high-risk group was associated with a poor prognosis and that the low-risk group had greater gene mutation rates than the higher-risk group. Since stromal cells are thought to reflect genetic stability and may be a target for reducing tumor resistance and recurrence, as shown in Figure 8B, the StromalScore and ESTIMATEScore scores of the high-risk group were substantially higher than those of the low-risk group (Quail and Joyce, 2013). Positive recommendations for extra treatment in the high-risk category follow as a result of this. We also found a strong relationship between risk scores and macrophage M0, which is implicated in tumor invasion and metastasis (Overacre-Delgoffe and Vignali, 2018), and Tregs cells, which are known to be effective inhibitors of anti-tumor immunity (Cassetta and Pollard, 2018). When we analyzed the high and low risk groups of immune-related genes, we found that the majority of the genes showed significant differences. We discovered that Paclitaxel and Imatinib were more sensitive in the low-risk group whereas Cisplatin and Metformin were more sensitive in the high-risk group, helping us to better understand the effects of our prognostic model on immunotherapy and drug sensitivity. As a result, we might infer that lysosome-related genes are important contributors to the tumor immunological environment and appealing immunotherapy targets. We looked at the connection between the model and clinical staging because we all know that clinical TNM staging, as an internationally accepted standard, has guiding value for the treatment and prognosis of tumor patients. We discovered a connection between the high-risk group and a higher Stage and TNM stage, which helped us understand why they had a worse prognosis.

Our research had certain limitations, of course, despite our thorough analysis of LRGs and COAD. The majority of prospective research, *in vitro* experiments, and *in vivo* tests are necessary to validate our findings because all of the investigations we performed utilizing the public database were retrospective (Jiang et al., 2016). Due to a paucity of data in this investigation, it was not possible to analyze other clinical variables, including whether surgery, neoadjuvant chemotherapy, and postoperative chemotherapy were carried out.

## Conclusion

In conclusion, our study found two separate subtypes of PLRGs in COAD, and the prognosis and TME of these two subtypes were noticeably different. Following that, the PLRGs_Score created using DEGs between the two subtypes had significant clinical importance and may offer recommendations for each patient’s subsequent treatment. Also, we were able to confirm for the first time that COAD had a highly MOGS expression.

## Data Availability

The datasets presented in this study can be found in online repositories. The names of the repository/repositories and accession number(s) can be found in the article/supplementary material.

## Supplementary material

SUPPLEMENTARY FIGURE S1 Subtypes and DFS analysis. **(A-H)** Consensus matrix heatmaps of PLRGs for *k* = 2-8. **(I)** Differences in DFS were compared between the two clusters using KM analysis.

SUPPLEMENTARY FIGURE S2 The AUC values of stage and TNM for 1-, 3- and 5- years. **(A-C) **The AUC values of TNM stage. **(D)** The AUC values of stage.

SUPPLEMENTARY FIGURE S3 Differences in clinical information among different PLRGs_score groups. **(A) **The age difference between the two groups (*p* = 0.26). **(B)** Gender differences between the two groups (*p* = 0.22).

SUPPLEMENTARY TABLE S1 Clinical data of 588 COAD patients.

SUPPLEMENTARY TABLE S2 28 prognostic lysosome-related genes.

SUPPLEMENTARY TABLE S3 2,129 differential genes between the two subtypes (log FC >= 1).

SUPPLEMENTARY TABLE S4 23 prognostic genes obtained from DEGs of the two subtypes using univariable cox analysis *(p* < 0.001).

SUPPLEMENTARY TABLE S5 7 genes were achieved by lasso regression and multivariable cox analysis.

## Abbreviations

LRGs, lysosome-related gene; COAD, colon adenocarcinoma; PLRGs, lysosome-related genes associated with prognosis; TME, tumor microenvironment; LASSO, least absolute shrinkage and selection operator regression; TCGA, The Cancer Genome Atlas; GEO, Gene Expression Omnibus; OS, overall survival; TMB, tumor mutation burden; CSC, cancer stem cell; MSS, microsatellite stability; DEGs, differentially expressed genes; FPKM, fragments per kilobase million; GO, Gene Ontology; CNV, copy number variation; KM, Kaplan-Meier; DFS, disease-free survival; ssGSEA, single-sample gene set enrichment analysis; GSVA, gene set variation analysis; MSI, microsatellite instability; MAF, mutation annotation format; IPS, immunophenotype score; NC, nitrocellulose; IHC, immunohistochemistry; SD, standard deviations; ROC, receiver operating characteristic; Tregs, regulatory T cells.
