# Supplementary figures and images for: Subtypes analysis and prognostic model construction based on lysosome-related genes in colon adenocarcinoma
Source: Front Genet. 2023 Apr 24;14:1149995. doi: 10.3389/fgene.2023.1149995 (PMC10166181; doi:10.3389/fgene.2023.1149995)

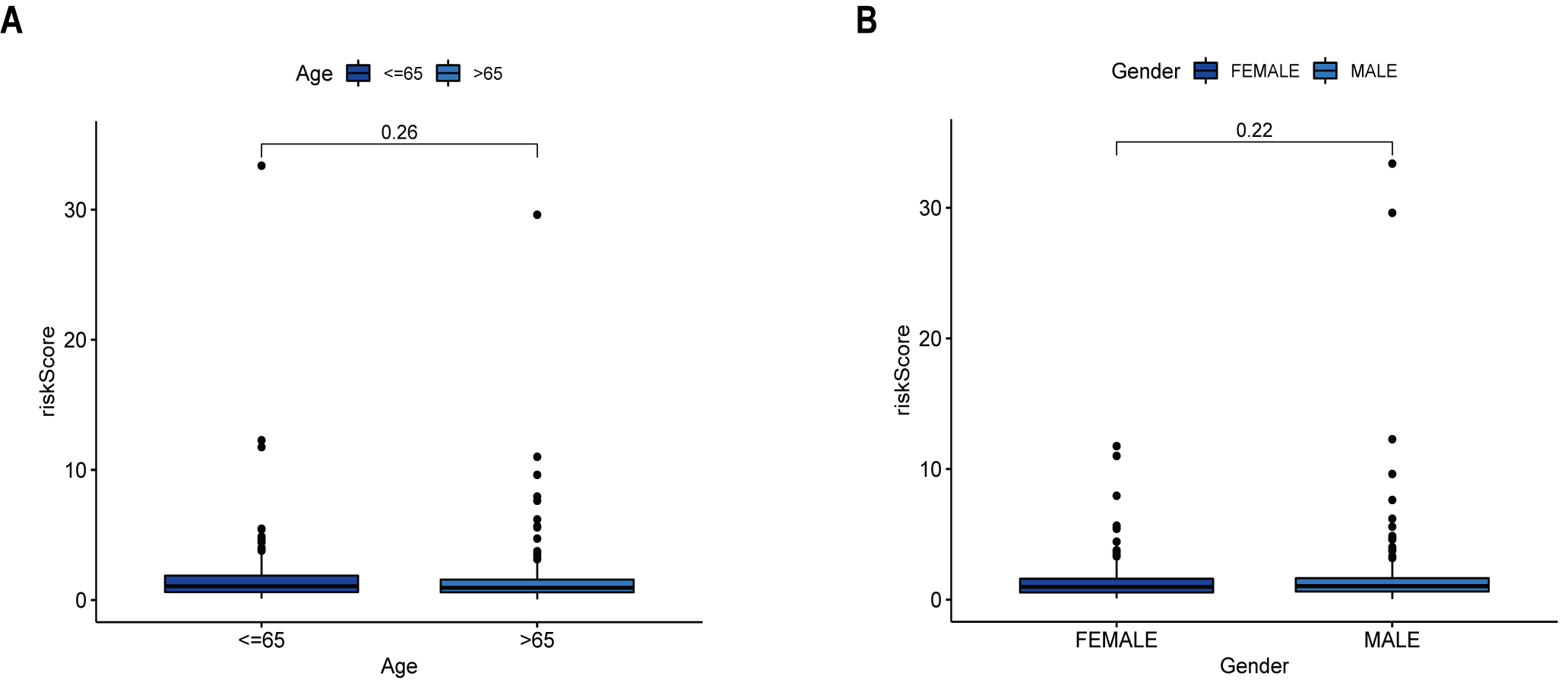

Supplement: Supplementary file 1 [file Image3.TIF]

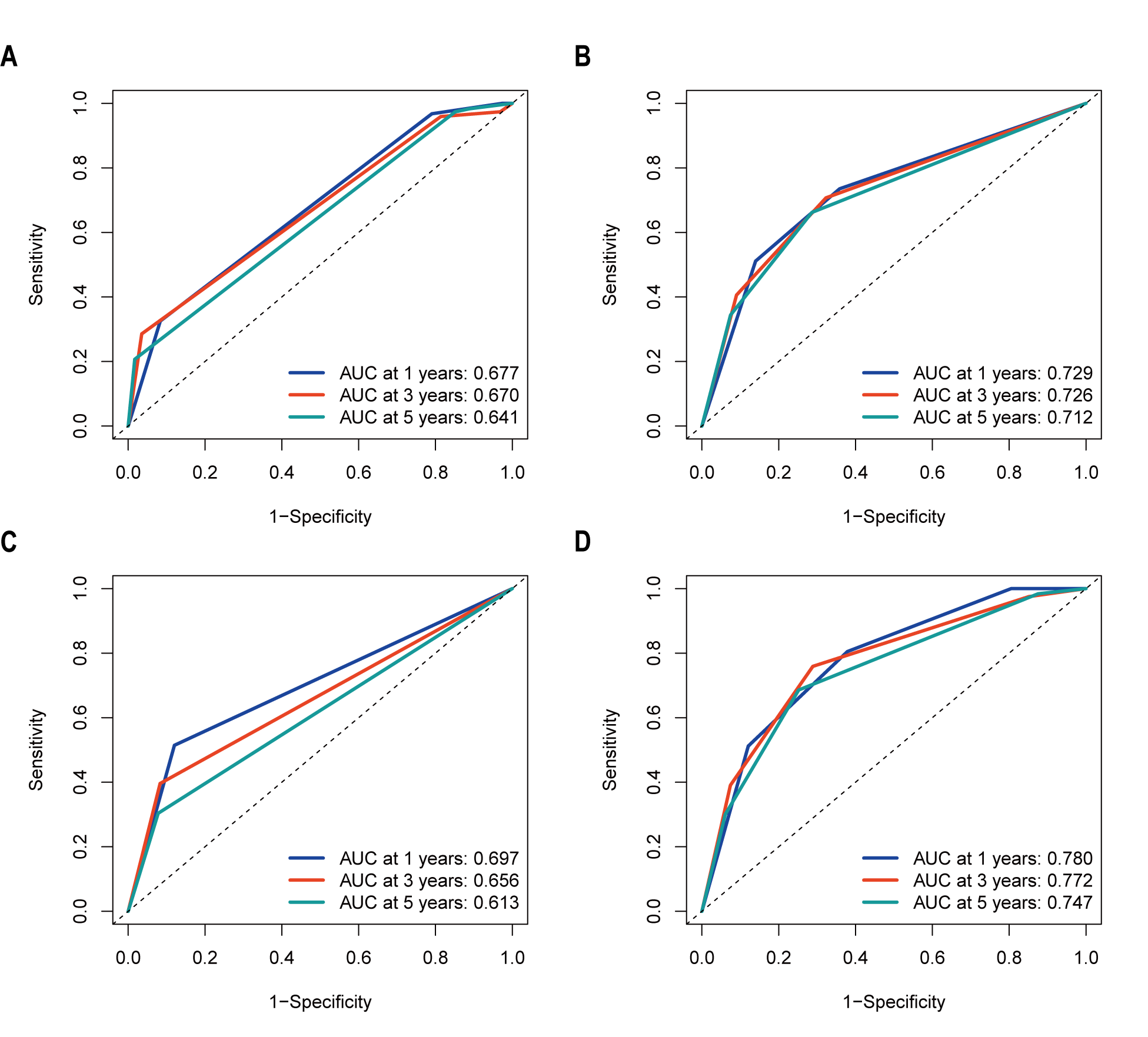

Supplement: Supplementary file 2 [file Image2.TIF]

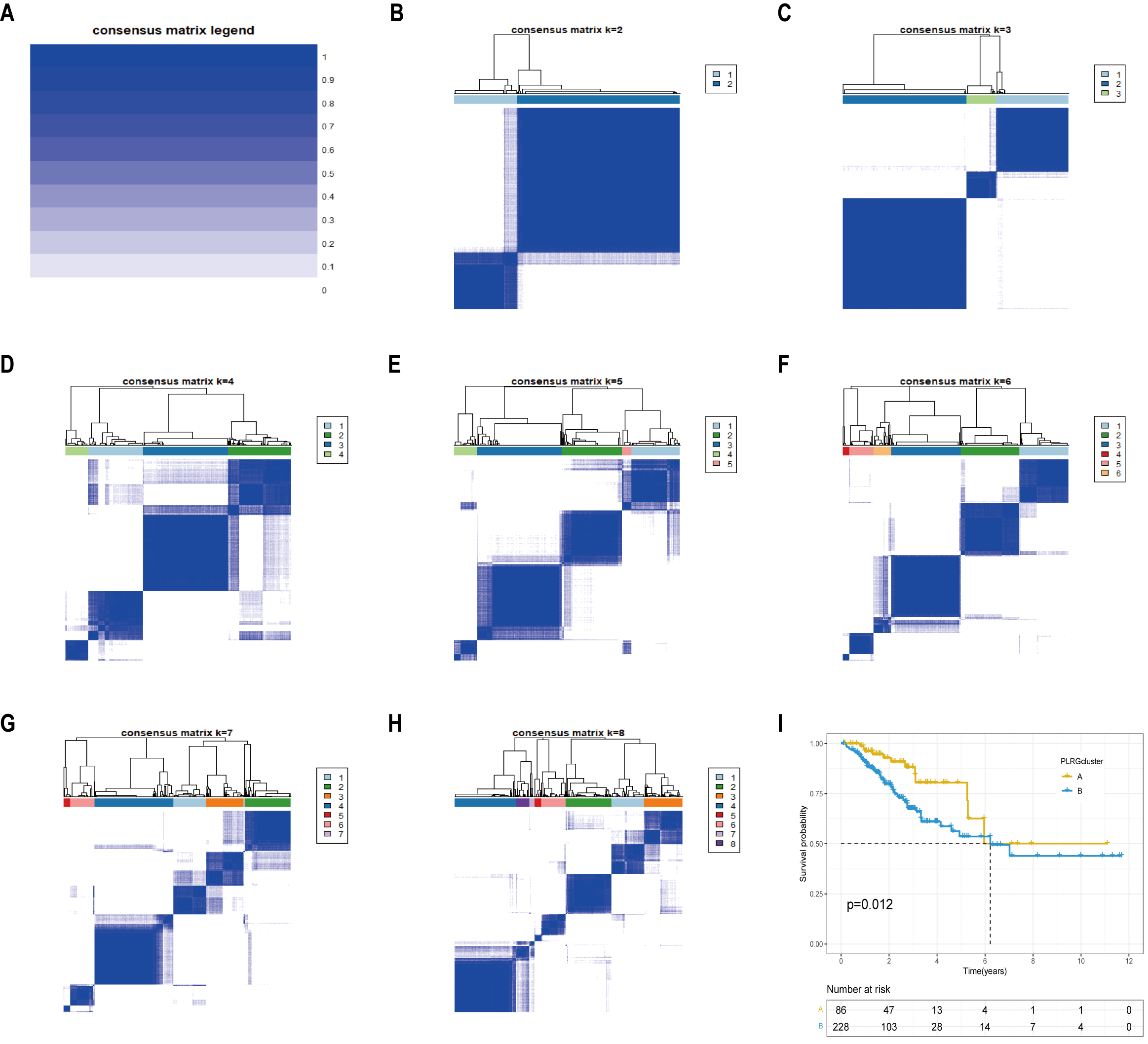

Supplement: Supplementary file 3 [file Image1.TIF]
